# Safety evaluation of Balanced Health Care Dan—A medicinal formulation containing traditional edible ingredients in lung tumor‐loaded mice

**DOI:** 10.1002/fsn3.3195

**Published:** 2022-12-21

**Authors:** Feng Dong, Changhui Zhao, Xiaoyun He, Yueyang Dong, Haiyan Liu, Peng Yao, Wentao Xu

**Affiliations:** ^1^ Institute of Acupuncture and Moxibustion China Academy of Chinese Medical Sciences Beijing China; ^2^ College of Food Science and Engineering Jilin University Changchun China; ^3^ College of Food Science and Nutritional Engineering China Agricultural University Beijing China; ^4^ Langfang Health Vocational College Langfang China; ^5^ Tianjin University of Sport Tianjin China; ^6^ Key Laboratory of Precision Nutrition and Food Quality Department of Nutrition and Health China Agricultural University Beijing China

**Keywords:** cancer, medicinal food, safety evaluation, toxicological assessment

## Abstract

Chinese formulation‐based medicinal food has been widely used in clinical trials, but its safety is not well studied. In this research, the edible safety assessment of Balanced Health Care Dan—a formulation containing traditional edible ingredients that were initially formulated to reduce side effects for lung cancer patients—was studied in mice based on biochemical and gut microbial analyses. The experimental mice were subcutaneously loaded with lung tumor A549 cells and then administrated with Balanced Health Care Dan (200 mg/kg or 400 mg/kg b.w. in gavage feeding) for 4 weeks. The body weight, blood parameters, and pathogenic phenotype in tissues were examined. No toxicological symptom was found in experimental mice compared with the normal control. Comprehensive analyses were also conducted to evaluate intestinal microbiota that are associated with many diseases. Balanced Health Care Dan modified the gut microbiota structure in a positive way. In conclusion, the Chinese formulation‐based medicinal food has shown no toxicological effect in mice within 4 weeks of feeding experiment and has the potential to be used in clinical trials.

## Graphical abstract

Chinese formulation‐based medicinal food has been widely used in clinical trials, but its safety is not well studied. The current research evaluated a medicinal food for cancer patients using biochemical and gut microbial analyses to support the use of such a formula.

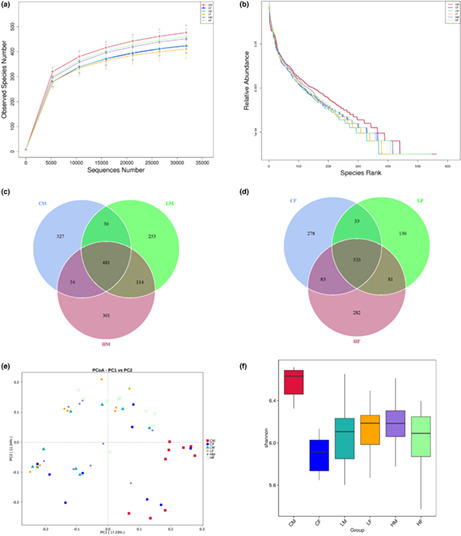

## 1 INTRODUCTION

Chinese formulation‐based medicinal food has made great progress in clinical uses. There are increasing varieties of traditional Chinese formulations used in cancer treatment, but the safety of these formulations has not been systematically evaluated.

Tumors are seriously threatening human life and health, leading to increased mortality and morbidity worldwide (Ferlay et al., 2021; Siegel et al., 2019). Among all diseases, cancer has the second highest death rate, only next to cardiovascular and cerebrovascular diseases (Siegel et al., 2018; Zhang et al., 2021). Lung cancer is a malignant tumor characterized by a rapid proliferation rate, less survivability, and high mortality. Lung cancer has the highest mortality rate among all cancers (Su et al., 2021). Surgery, radiotherapy, and chemotherapy are the most common clinical treatment strategies (Couzin‐Frankel, 2013; Ma et al., 2019). These treatments have greatly improved the prognosis of lung cancer patients, but also have brought about many side effects (Wang et al., 2020, 2021). These side effects greatly reduce the quality of life of the patients during treatment.

Traditional medicinal food (TMF) offers a promising option to reduce the side effects during cancer treatment (Luo et al., 2019; Zhang et al., 2022). “Balanced Health Care Dan” is a formula that is designed to improve patient's quality of life, and decrease chemotherapy‐induced adverse effects. Although most of the TMFs are botanical and have been traditionally considered to be nontoxic, the ingredients of TMF are generally complex with certain substances that might cause additive toxicities (Lin et al., 2018; Zhang & Yuan, 2012). Therefore, edible safety evaluation and clinical studies of TMFs are equally important. Animal‐based toxicity evaluation is necessary before the clinical application of these TMFs added to the diet of cancer patients (Shen et al., 2014; Wang, 2015). The current research focuses on the edible safety evaluation of “Balanced Health Care Dan,” which can be considered a model for traditional formula‐based medicinal food.

## 2 MATERIALS AND METHODS

### 2.1 Animals and cells

Totally, 72 BALB/C Nude mice (36 males and 36 females) of 5‐week‐old with an average body weight of 40–60 g were purchased from Vital River Laboratory Animal Technology Co., Ltd. The animal room was maintained at 23 ± 2°C, with relative humidity of 50 ± 5%. A 12 h light/dark cycle was provided by automated fluorescent illumination. All mice were provided with their diet and water ad libitum. The animal studies were approved by the Animal Care and Use Committee at China Agricultural University and all experiments were performed in accordance with relevant guidelines and regulations (Approval Number: AW02110202‐4).

A549 lung cancer cells (ATCC) were cultured in a carbon dioxide cell incubator at 37°C. The medium was Dulbecco's modified Eagle medium basal medium, complemented with 10% fetal bovine serum and 100 U/ml penicillin and streptomycin. The cells were digested with 0.25% trypsin and passaged on alternate days.

### 2.2 Traditional formula ingredients

The Balanced Health Care Dan was prepared using the following ingredients: Dangshen, *Astragalus membranaceus*, *Atractylodes macrocephala*, white lentil, *Ligusticum chuanxiong*, bezoar, musk, Rhodiola, *Platycodon grandiflorum*, mulberry bark, licorice, *Poria cocos*, wood incense, *Sichuan pepper*, aloes, polygonatum, purple *Ganoderma lucidum*, *Hedyotis diffusa*, *Dendrobii Officmalis* Caulis, pollen, and honey. Each ingredient was washed, dried, disinfected, and ground into very fine powder. The powder was then mixed with condensed honey followed by pellet making for serving. Currently, the formulation is in the application for patent protection (Application No. CN20180374829.6).

### 2.3 Mouse lung cancer tumor modeling

Each mouse was subcutaneously inoculated with A549 cells (5 × 10^6^ cells/mouse) on the right back. The animals were used for the experiment when the average tumor volume of the group reached over 100 mm^3^. The successful model mice were randomly divided into groups as follows: Control model mice were gavaged with sterile water, while low‐dose group and high‐dose groups of mice were gavaged with 200 or 400 mg/kg of Balanced Health Care Dan solution for 4 weeks, respectively. The doses used were physiologically relevant to that administered to humans. The treatment was carried out in six consecutive days per week. The daily body weight of the mice was recorded.

### 2.4 Blood biochemistry

At the end of the experiment, mice were fasted for 12 h before blood collection. Blood samples were collected from the inner canthus under anesthesia. The serum samples were analyzed for alkaline phosphatase (ALP), aspartate aminotransferase (AST), alanine aminotransferase (ALT), albumin (ALB), creatinine (CREA), urea, triglycerides (TG), and cholesterol (CHO) as previously reported (He et al., 2020).

### 2.5 Pathology examination

The fresh tissues of the tumor, liver, kidney, spleen, and lung of mice were fixed with 4% paraformaldehyde for 24–48 h. The tissues were dehydrated, waxed, embedded, and sliced. Subsequent HE staining was performed and then examined microscopically by a professional staff.

### 2.6 Fecal metagenomic analysis

The intestinal contents of mice were collected in sterilized tubes and frozen at −80°C. DNA was then extracted from the intestinal contents according to the instructions in the kit (FDA6512, Beijing Ford Press Technology Co., LTD). The 16S rDNA sequencing and data analysis were performed as reported (He et al., 2020; Xu et al., 2020). Briefly, The V3‐V4 region of the 16S rDNA was amplified by PCR with specific primers linked to the barcode. Thermal cycling consisted of initial denaturation at 98°C for 1 min, followed by 30 cycles of denaturation at 98°C for 10 s, annealing at 50°C for 30 s, and elongation at 72°C for 30 s. Finally, 72°C for 5 min. Sequencing libraries were generated using TruSeq® DNA PCR‐Free Sample Preparation Kit (Illumina) following the manufacturer's recommendations, and index codes were added. The library quality was assessed on the Qubit@2.0 Fluorometer (Thermo Scientific) and Agilent Bioanalyzer 2100 system. At last, the library was sequenced on an Illumina NovaSeq platform. After data filtering, UPARSE software (uparsev7.0.1001) was used to cluster valid data into Operational Taxonomic Units. Mothur method and SILVA138 (http://www.arb‐silva.de/)'s SSUrRNA database were used for species annotation analysis. Qiime software (Version 1.9.1) was used to analyze diversity. R software (Version 2.15.3) was used for PCA analysis. LEfSe software was used for LEfSe analysis, and the filter value of LDA Score was 4 by default (Segata et al., 2011).

### 2.7 Statistical analysis

The experimental data were presented as mean value ± standard deviation, and the data were analyzed by GraphPad Prism 8. A one‐way analysis of variance (ANOVA) was applied with Dunnet‐1 post hoc analysis. Differences between values were considered statistically significant at *p < .05, and extremely significant at **p < .01.

## 3 RESULTS AND DISCUSSION

### 3.1 Tumor incidence

**FIGURE 1:**
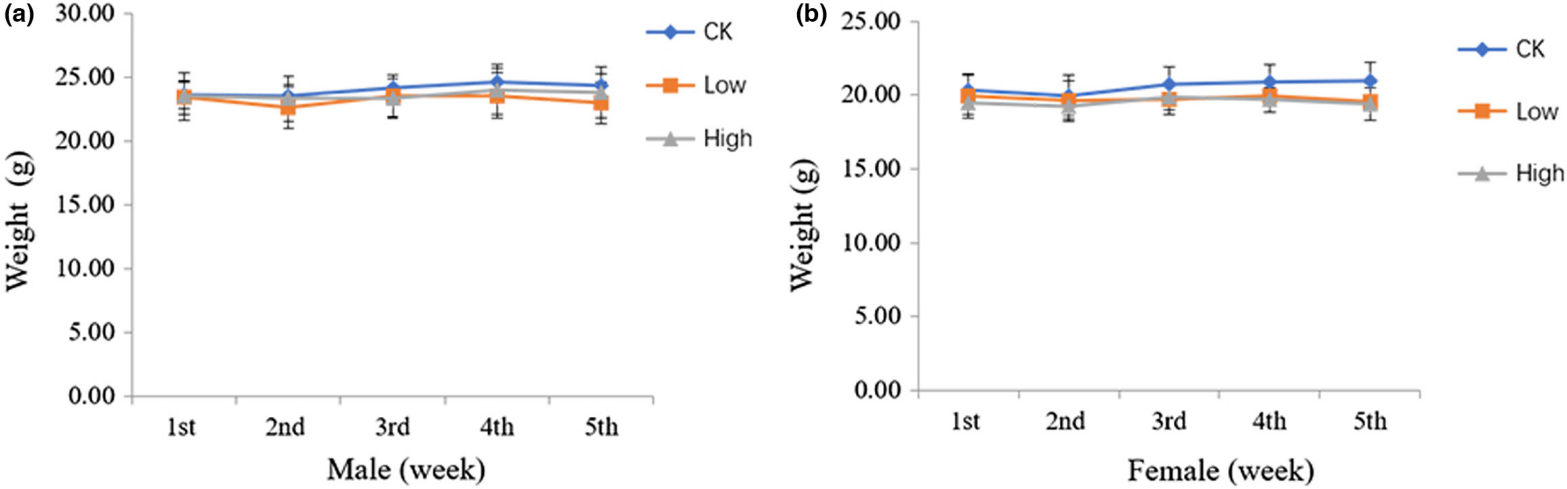
Body weight of male and female mice during treatment. (a) Male mice; (b) female mice. CK, control group; Low, low‐dose group; High, high‐dose group

A total of 72 mice (36 female and 36 male mice) were used in this experiment, among which 54 mice (27 males and 27 females) were successfully loaded with tumors that met the requirement and were included in the follow‐up experiment. The 54 mice were randomly divided into six groups (3 male groups and 3 female groups; 9 mice/group) following a computerized randomization scheme based on body weight. Four weeks treatment with Balanced Health Care Dan did not significantly affect the body weight of these mice (Figure 1a).

### 3.2 Analysis of clinical appearance

During the 4‐week experiment, no treatment‐related adverse effects in the clinical appearance of the animals were observed. The body weight of male and female mice in the experimental groups was comparable with that of the control group on day 7, day 14, day 21, and at the end of the experiment. Balanced Health Care Dan slightly increased body weight nonsignificantly, demonstrating that the formula did not exhibit any acute toxicity effects on the animals' growth and development (Hamaguchi et al., 2019).

### 3.3 Analysis of hematology

**FIGURE 2:**
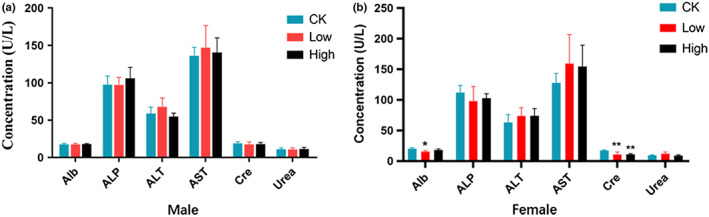
Blood biochemistry of male and female mice during treatment. (a) Male mice; (b) female mice

**FIGURE 3:**
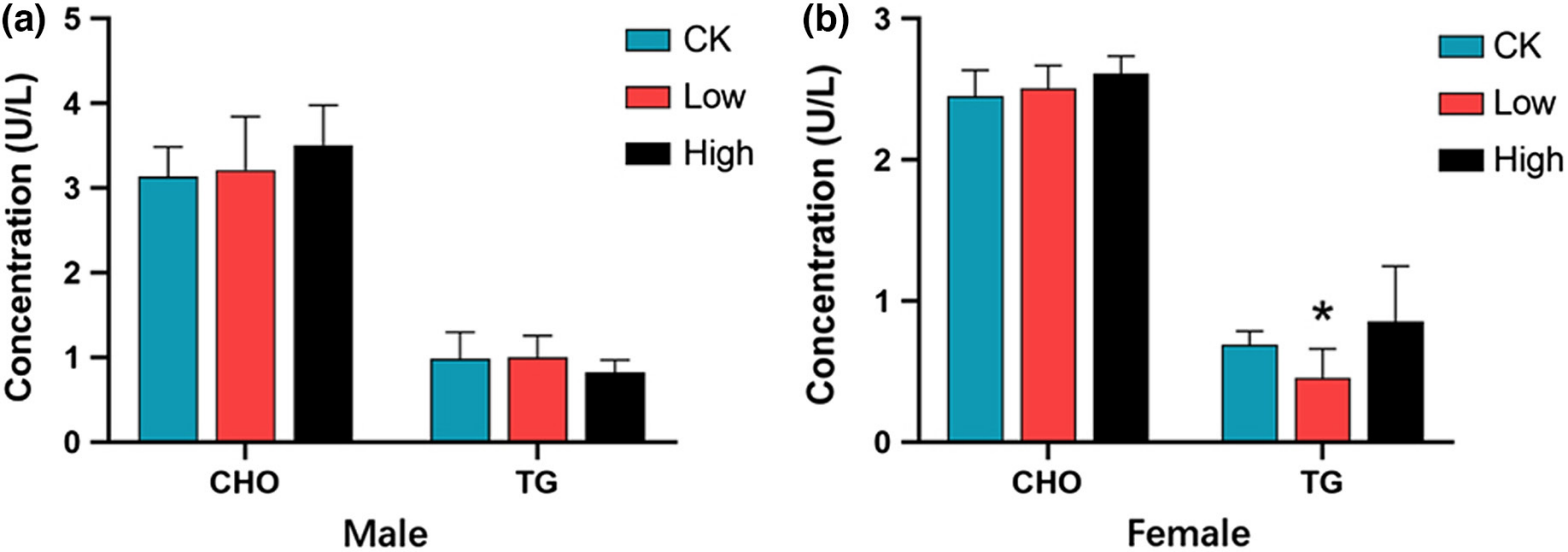
Lipid profile of male and female mice during treatment. (a) Male mice; (b) female mice. CHO, cholesterol; TG, total triglycerides

Hematology index is an important indicator in safety evaluation (Rosa et al., 2018). Serum biochemical profile derangement can reflect nutrient metabolism abnormality (Ca Llens & Bartges, 2015; Gwinn et al., 2020; Paiano et al., 2019), as damaged tissues or organs modify the serum parameters. We detected blood biochemical indexes including ALB, ALP, ALT, AST, CREA, Urea, CHO, and TG at day 28 (Figures 2 and 3). These indicators can reflect liver, kidney function, and lipid metabolism. Liver and kidney are important target organs of many toxins, which can alter relevant indicators after intragastric administration (Calle‐Toro et al., 2020; Ursell et al., 2012). As the important indexes of liver and kidney function, the values of ALP, ALT, AST, and Urea were not significantly different between groups. In female groups, the mean values of ALB and CREA were slightly lower in low‐dose and high‐dose groups, respectively, compared with control. However, these differences were within the normal range, which possibly resulted from individual differences between mice. Such differences were not seen in male mice.

The lipid profile reflects basic metabolism of the body. CHO and TG are the common indicators for lipid metabolism. In the female group, the mean value of TG in the low‐dose group reduced slightly compared with that of the control group indicating the treatment might have a hypolipidemic effect. Since this beneficial effect was not shown in the high‐dose group, we think the difference was more likely attributed to the background variability and sporadic deviation.

### 3.4 Analysis of pathology in organs

A complete gross necropsy and microscopic anatomic pathological analysis of organs were conducted on all animals after the 4‐week feeding study. The liver, kidney, lung, and spleen showed no pathological lesions (data not shown) in the low‐dose or high‐dose groups. From the pathological point of view, Balanced Health Care Dan does not have any toxic or adverse effects.

### 3.5 Analysis of microbiota

Intestinal flora is known as the “second fingerprint” of the body (Duffy et al., 2015; Ursell et al., 2012), which has an enormous impact on the nutrition and health status of the host. Diet directly affects the balance of intestinal microbiota. For edible safety evaluation, analyzing the changes in intestinal microbiota can directly reflect the effects of tested materials on body health (Barko et al., 2017). Therefore, the evaluation of intestinal microbiota is an important part of edible safety evaluation. Thus, in this study, the intestinal contents were used to evaluate the effects of Balanced Health Care Dan.

**FIGURE 4:**
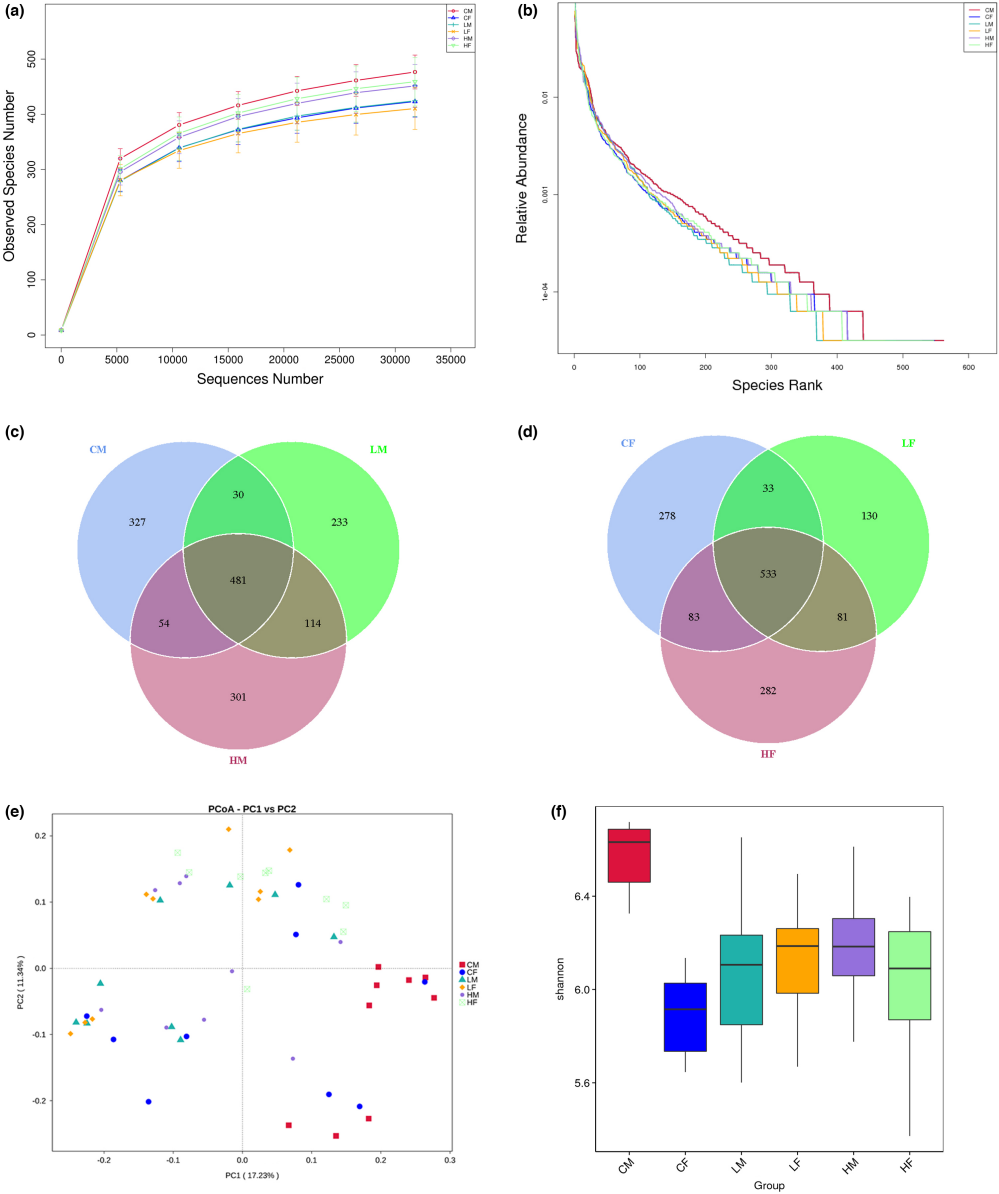
The overall level of intestinal flora change. (a, b) The overall level of intestinal flora changed; (c) OUT analysis of each dose group in male mice; (d) OUT analysis of each dose group in female mice; (e) principal coordinate analysis of male mouse samples; (f) principal component analysis of female mouse samples

Based on metagenomic sequencing, in both male and female mice, the relative abundance of intestinal microbiota changed after TMF treatment (Figure 4a,b). Principal coordinates analysis showed significant changes in the intestinal microbiota of male mice in the TMF‐treated group compared with the control group, while the effect of TMF on the intestinal microbiota of female mice was relatively small (Figure 4c,d). The Shannon index was used to measure species diversity (Nielsen, 2021). From Figure 4e,f, TMF treatment significantly altered the structure and composition of intestinal microbiota in both male and female mice.

**FIGURE 5:**
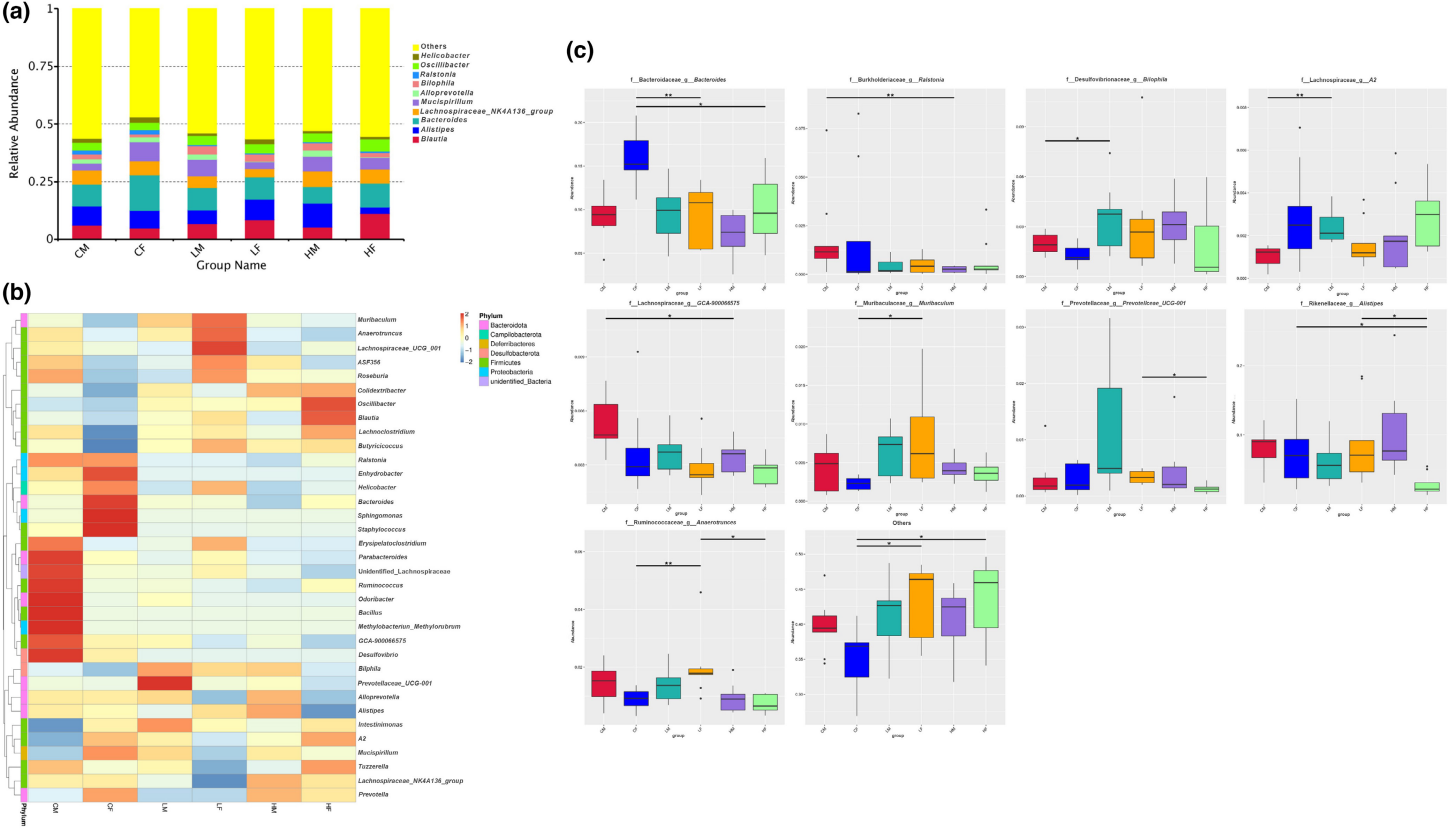
Changes in the level of intestinal flora. (a) Top 10 dominant bacteria analysis; (b) top 35 dominant bacteria thermogram analysis; (c) abundance distribution box map between groups

**FIGURE 6:**
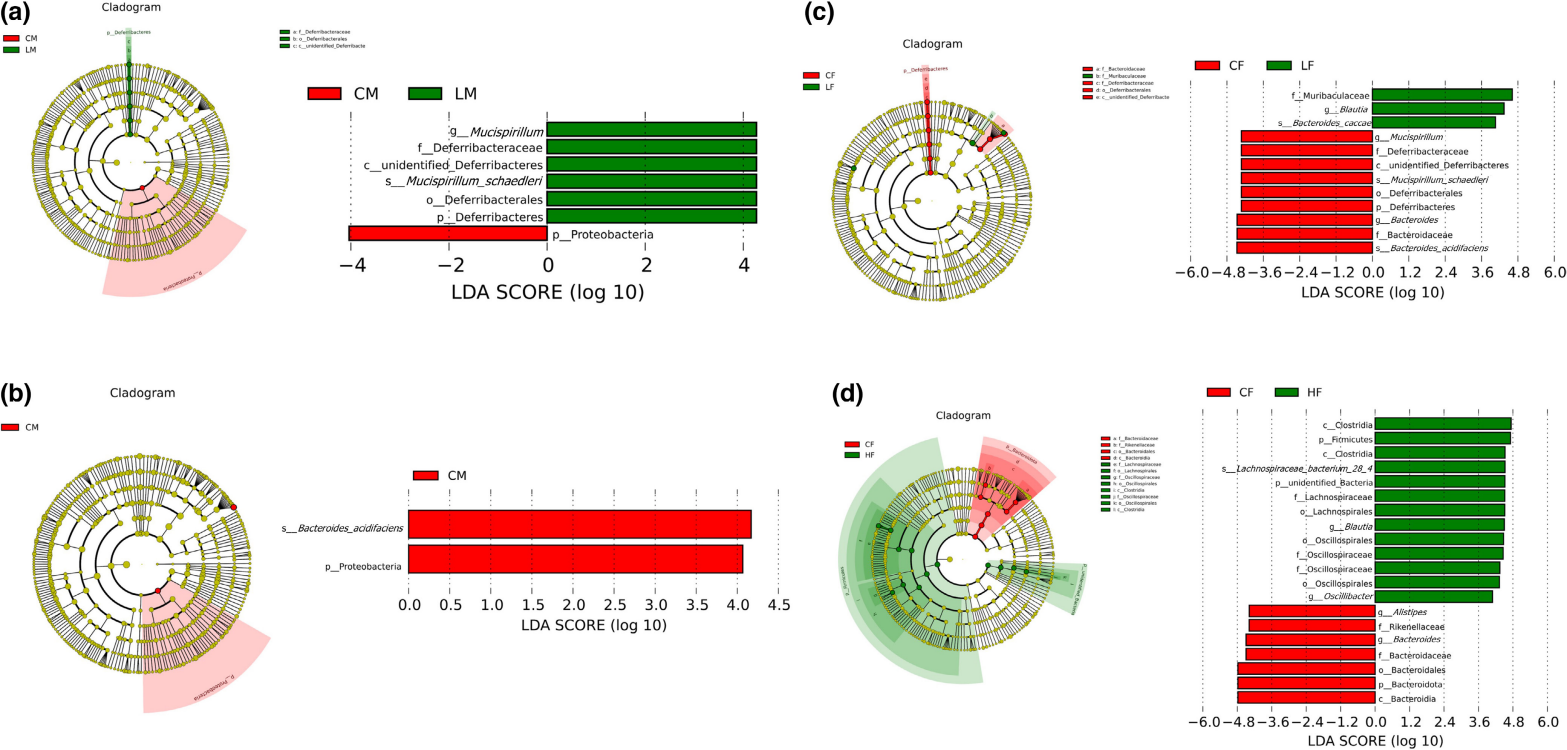
LEfSe analysis in mice (a) and (b) Male mice; (c) and (d) female mice.

**FIGURE 7:**
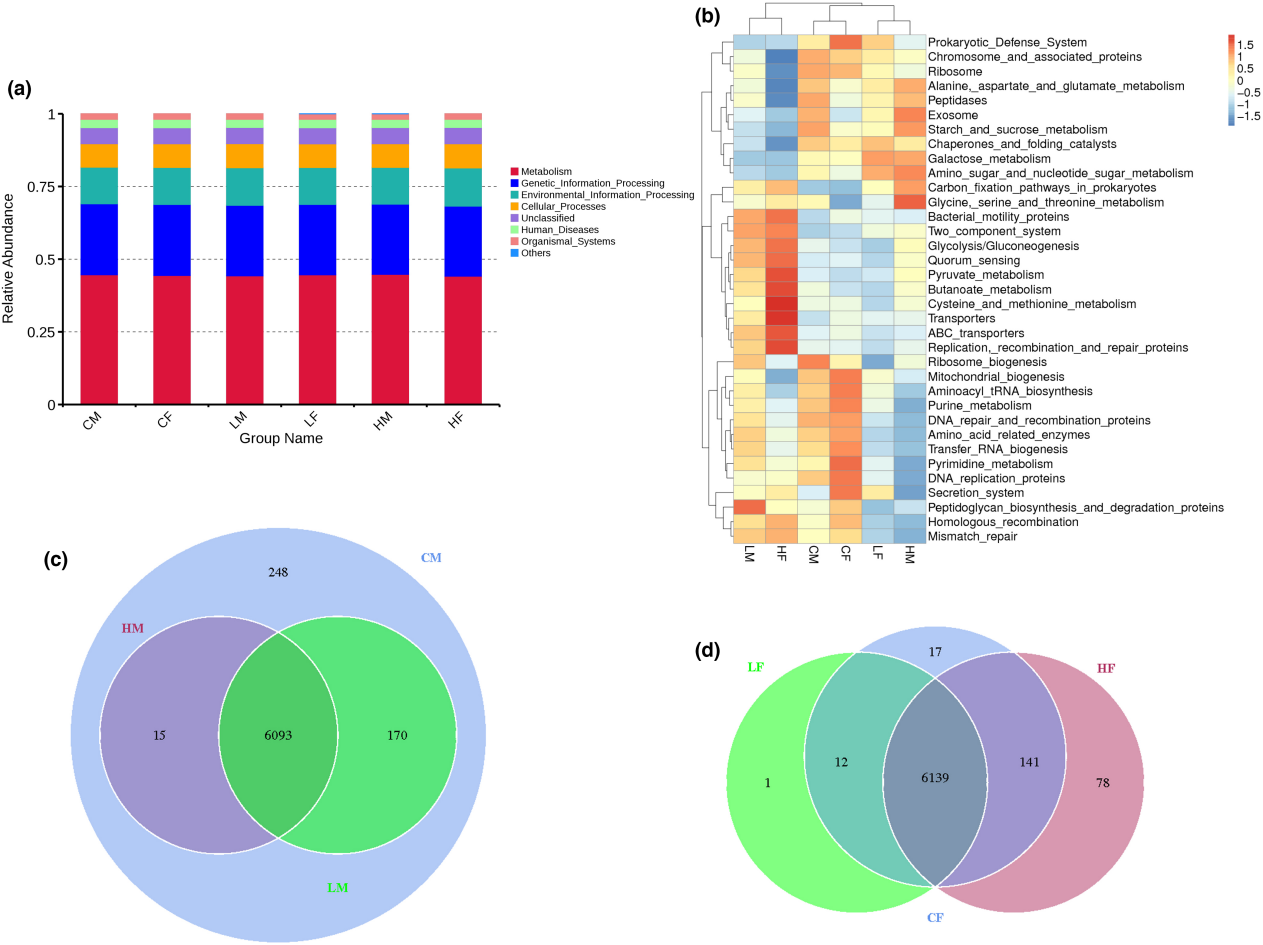
Function prediction analysis. (a) Relative abundance analysis of top 10 function annotation; (b) cluster analysis of relative abundance of top 35 function; (c) functional notes of Venn diagram; (d) function annotation of petal diagram

From the analysis of the genus level, the most predominant 10 genera were identified (Figure 5a). According to the species annotation and abundance information of all samples at the genus level, correlation heatmap was applied to represent the top 35 genera (Figure 5b). Compared with the control group, *Bacteroides*, *Ralstonia*, *Bilophila*, *Muribaculum*, *Prevotellaceae*, *Alistipes*, and *Anaerotruncus* all showed significant changes in the TMF treatment groups (Figure 5c). The results showed that there were different effects on the mice by gender. For example, in male mice groups as shown in Figure 6a,b, *Deterribacteres*, *Deferribacteraceae*, *Deferribacterales*, and *Deferribacteres* increased significantly compared with the control group. B*acteroides acidifaciens* and *Proteobacteria* decreased significantly. In addition, the proportion of *Firmicutes* to *Bacteroidetes* did not change significantly. While in the female mice groups as shown in Figure 6c,d, *Muribaculaceae*, *Blautia*, *Bacteroides caccae*, *Clostridia*, *Firmicutes*, *Lachnospiraceae Bacterium*, *Lachnospiraceae*, *Oscillibacter*, *Lachnospirales*, *Oscillospirales*, and *Osillospiraceae* increased significantly. *Mucispirillum*, *Deferribacteraceae*, *Deferribacterales*, *Deferribacteres*, *Bacteroides*, *Alistipes Bacteroidaceae*, *Bacteroides acidifaciens*, *Bacteroidales*, *Bacteroidota*, *Bacteroidia*, and *Rikenellaceae* significantly decreased. In addition, the proportion of *Firmicutes* to *Bacteroidetes* increased significantly. In order to further confirm the gender difference in the effects of TMF, functional predictive analysis was performed. As shown in Figure 7a, there was no difference in the top 10 functions between male and female mice although the function of the top 35 varied by gender (Figure 7b).

There was more *Bacteroides* in the gut of colorectal cancer patient, which indirectly proved that the decrease in *Bacteroides* had a positive effect on the body's resistance to cancer (Garrett, 2019). *Proteobacteria* have been found to dominate the intestinal microbiota in acute and chronic inflammation caused by infectious pathogens or protozoan parasites, and the same phenomenon has been found in colorectal cancer associated with enteritis in animal and human experiments (Da et al., 2020). Balanced Health Care Dan significantly reduced *Proteobacteria*, indicating that Balanced Health Care Dan could help improve body health. The abundance of *Muribaculaceae* was negatively correlated with proinflammatory factors (Chung et al., 2020), and the increasing abundance of *Muribaculaceae* in the study indicated that it could protect intestinal health. The abundance of *Lachnospiraceae* was increased in the TMF treatment groups. As a potentially beneficial bacterium, *Lachnospiraceae* participates in the metabolism of a variety of carbohydrates, among which acetic acid, the fermentation product, is the main source of energy for the host (Vacca et al., 2020). In contrast to the control groups, the proportion of *Firmicutes* to *Bacteroidetes* in male mice groups did not change but significantly increased in female mice group, suggesting that after high‐dose treatment, female mice were more likely to absorb heat to maintain body weight (John & Mullin, 2016; Zhao et al., 2021). Therefore, to a certain extent, Balanced Health Care Dan improved the intestinal microbiota of mice and then might increase body immunity. This effect was more obvious in male mice. Therefore, from the point of view of intestinal health, Balanced Health Care Dan is not potentially harmful to the body. It needs to be noted that there are huge differences in gut microbiota between human and animal models, which are caused by species‐specific differences, evidenced by host–microbial interactions, environment, diet, and genetic responses (Nguyen et al., 2015). Further clinical trial is still necessary for accurate risk assessment.

## 4 CONCLUSION

In a 4‐week animal feeding test, we evaluated edible safety of a traditional formula‐based medicinal food called Balanced Health Care Dan by examining behavior performance, body weight, relevant blood parameters, pathological phenotype, and intestinal microbiota in lung tumor‐loaded mice. We found no treatment‐related adverse effects in this short‐term toxicity evaluation experiment. This research provides a new strategy for the edible safety evaluation of TMFs which have the potential to be used for cancer patients.

## CONFLICT OF INTEREST

The authors declare no conflict of interest in this study.

## Data Availability

The datasets used and/or analyzed during this study are available from the corresponding author upon reasonable request.
